# Rapid, quantitative, and high-sensitivity detection of anti-phospholipase A2 receptor antibodies using a novel CdSe/ZnS-based fluorescence immunosorbent assay

**DOI:** 10.1038/s41598-021-88343-z

**Published:** 2021-04-22

**Authors:** Chenxi Li, Manyun Qian, Qiaozhen Hong, Xiaohong Xin, Zichun Sun, Yafeng Li, Bo Tang, Bing Gu

**Affiliations:** 1 Xuzhou Key Laboratory of Laboratory Diagnostics, Medical Technology School of Xuzhou Medical University, Xuzhou, 221004 China; 2 Department of Nephrology, The Shanxi People’s Hospital, Shanxi Medical University, Taiyuan, 030001 Shanxi China; 3 Department of Laboratory Medicine, Quzhou Kecheng People’s Hospital, Quzhou, 324000 China; 4 Nanjing Vazyme Medical Technology Co. Ltd., Nanjing, 210046 China; 5 Department of Laboratory Medicine, The Affiliated Hospital of Xuzhou Medical University, Xuzhou, 221006 China

**Keywords:** Immunology, Autoimmunity

## Abstract

Autoantibodies against M-type phospholipase A2 receptor (PLA2R) serve as specific biomarkers for idiopathic membranous nephropathy (IMN), and its quantification helps monitor disease activity. In this study, we describe a rapid and highly sensitive quantum dots-based immunochromatography assay (QD-ICA) for quantifying PLA2R autoantibodies. Serum samples from 135 biopsy-confirmed patients with nephrotic syndrome were analyzed for PLA2R autoantibodies using the novel QD-ICA as well as commercialized enzyme-linked immunosorbent assay (ELISA). Areas under the receiver operating characteristic curve (AUC-ROC) of QD-ICA were significantly greater than those of ELISA (91.1% [95% CI 85.9–96.3%] and 83.9% [95% CI 76.5–91.2%] respectively; p < 0.01). The detection sensitivity and specificity of QD-ICA (80.9% [95% CI 69.2–89.0%] and 100% [95% CI 93.2–100.0%], respectively) exceeded those of ELISA (72.1% [95% CI 59.7–81.9%] and 98.5% [95% CI 90.9–100.0%], respectively). The optimum cut-off value of QD-ICA was 18.18 relative units (RU)/mL, and the limit of detection was 2.86 RU/mL. The novel QD-ICA outperforms ELISA in detecting PLA2R autoantibodies, with shorter detection time, fewer steps, smaller equipment size, and broader testing application, suggesting its capability to improve IMN diagnosis and monitor patient response to treatment.

## Introduction

Membranous nephropathy (MN), an organ-specific autoimmune disease, is one of the leading causes of nephrotic syndrome. Different forms of MN have been defined according to their pathogenesis: idiopathic MN (IMN) is detected in 80% of patients with MN and secondary MN (SMN) is related to various autoimmune diseases, infections, and malignancies^1^. Usually, MN exhibits an asymptomatic onset, and approximately 20% of the patients do not display proteinuria within the nephrotic range. MN develops slowly, and some patients experience spontaneous remission; however, in more than 30% of the cases, the disease eventually progresses to end-stage renal disease or leads to death^2,3^. Epidemiological studies reveal that the incidence of IMN accounted for 6.48% of primary glomerular cases from 1997 to 1999 and it increased to 22.7% from 2009 to 2011^4,5^, and the incidence has significantly increased in recent years. The M-type phospholipase A2 receptor (PLA2R) belongs to the mammalian mannose receptor family and is a biomarker of IMN that is mainly expressed in podocytes^6^. Studies suggest that the PLA2R antigen in IMN binds to its antibody to form an in situ immune complex, which activates the complement system to form the complement membrane attack complex, leading to podocyte injury^7,8^, resulting in a series of abnormalities of renal function indicators including proteinuria, hypoproteinemia, hypercoagulability, and metabolic disorders.

Currently, IMN diagnosis relies on invasive renal biopsy, followed by examination using light microscopy, electron microscopy, and/or immunostaining. Although a routine blood biochemistry test can confirm some cases with proteinuria, the infeasibility of performing an invasive renal biopsy in all suspected patients with MN results in delayed diagnosis. Studies have reported that circulating PLA2R autoantibodies are detectable in 52–82% of patients with IMN but are rare or absent in those with SMN^9–12^. The anti–PLA2R autoantibody titer in the serum is closely associated with the immunological activity and clinical status of patients with IMN^13,14^. This association between PLA2R autoantibodies and disease activity suggests that the monitoring and quantification of autoantibodies may be for therapy in patients with MN and a valuable tool for determining treatment strategies. In addition to PLA2R, podocyte biomarkers identified in patients with IMN include neural epidermal growth factor-like 1 protein (NELL-1) and thrombospondin type-1 domain-containing 7A (THSD7A), accounting for 5–10% and 3–5% of adult IMN, respectively^15^. Currently, only serum anti-PLA2R antibody detection is widely used in guiding the IMN clinical diagnosis, prognosis, and treatment of IMN.

The development of an anti-PLA2R antibody detection system has been a long process. Initially, Beck et al.^9^ successfully detected anti-PLA2R antibodies using nonreducing sodium dodecyl sulfate–polyacrylamide gel electrophoresis (SDS-PAGE) and western blotting. Although the sensitivity of detecting anti-PLA2R antibodies using western blotting is over 70%, it cannot be performed outside the laboratory, and testing many samples simultaneously is difficult. A recombinant HEK293 cell-based assay utilizing indirect immunofluorescence (CBA-IFA) was established, which was effective for IMN diagnosis and monitoring; however, it could evaluate anti-PLA2R antibody levels only semi-quantitatively^11^. Subsequently, an enzyme-linked immunosorbent assay (ELISA) that uses a recombinant PLA2R extracellular domain as a substrate for the quantitative detection of PLA2R in the serum of patients with IMN was developed^16^. However, in ELISA, signal generation is based on time-dependent enzymatic reactions, and enzymes are susceptible to environmental factors and highly vulnerable to the influence of matrix components^17^. Recently, addressable laser bead immunoassay (ALBIA)^18^, time-resolved fluoroimmunoassay (TRFIA)^19^, luciferase immunoprecipitation systems (LIPS)^20^, and chemiluminescence immunoassay (ChLIA)^21^ have been reported for the quantitative detection of anti-PLA2R antibodies. Although these methods have contributed significantly toward the detection of PLA2R antibodies, the detection equipment is not portable, and the detection is not sufficiently rapid. Hence, there remains a need to develop more rapid, convenient, and cost-effective point-of-care testing (POCT) detection systems for clinical laboratories.

**Figure 1:**
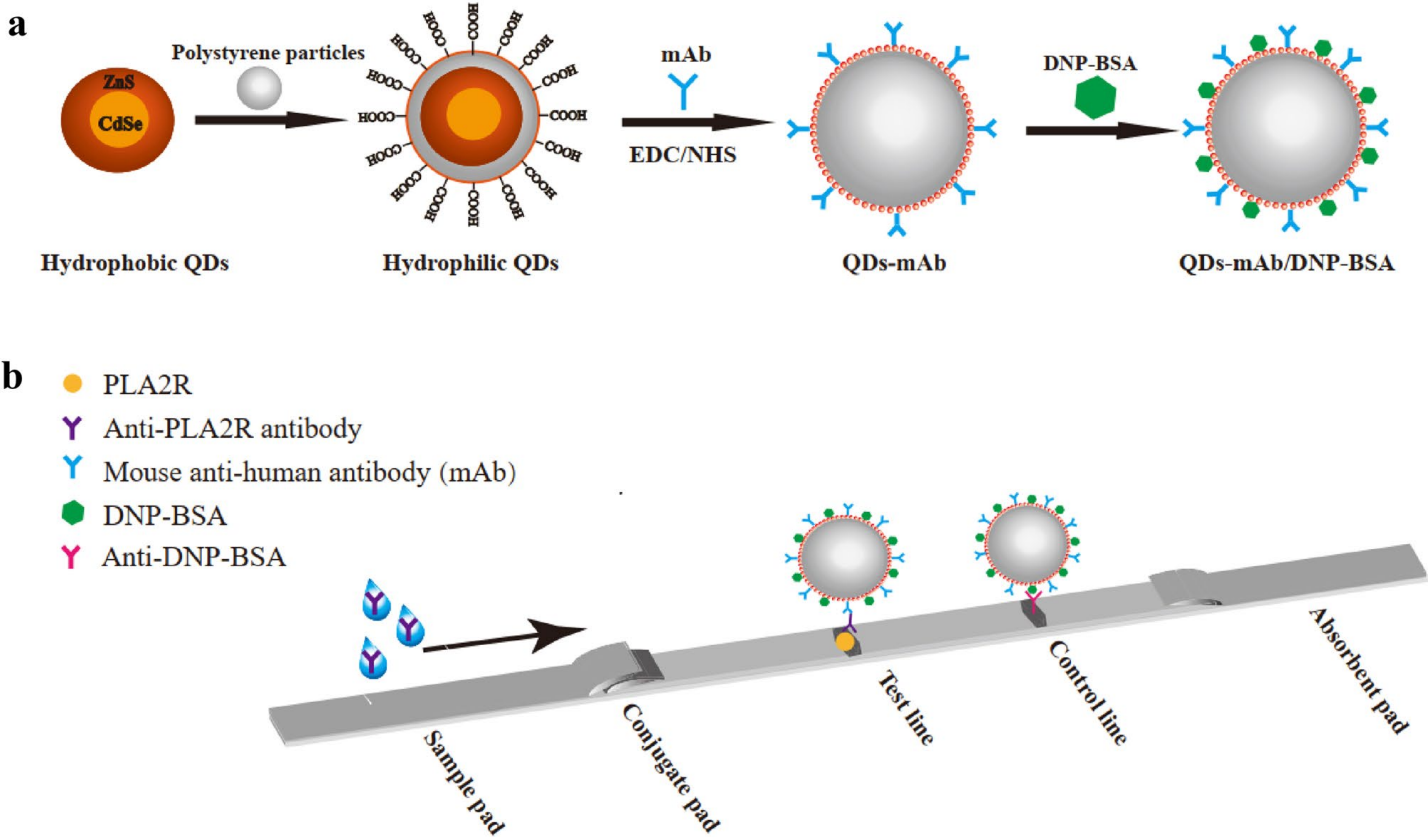
Quantum dot conjugate formation and antibody detection. Schemes used for (**a**) quantum dots–antibody conjugate synthesis and (**b**) identification of anti-PLA2R antibodies. *QDs* quantum dots, *mAb* monoclonal antibody, *DNP-BSA* N-2,4-dinitrophenylated-bull serum albumin.

Here, we describe a novel quantum dot-based immunochromatography assay (QD-ICA) for the quantitative detection of anti-PLA2R antibodies. This POCT assay uses an aqueous QD-based probe with CdSe as a core and ZnS as a shell conjugated with a monoclonal antibody (mAb) (Fig. 1). The novel assay not only decreased the detection time but also reduced the number of operation steps. Importantly, the assay demonstrated improved clinical sensitivity as well as specificity. Thus, the proposed assay has a great potential as a cost-effective and convenient in vitro nanomedicine-based diagnostic kit for IMN.

## Results

### Clinical characteristics of the patients

A total of 135 biopsy-confirmed patients with nephrotic syndrome (including 68 patients with IMN and 67 patients without IMN) who visited the Department of Nephrology, Shanxi Provincial People’s Hospital (Shanxi, China) were enrolled. The median age of all patients was 48 (interquartile range 36–57) years. The ratio of females to males was 1:1.2. There were significant differences in serum total protein levels, serum albumin levels, 24-h proteinuria, Cystatin C content, and estimated glomerular filtration rate (eGFR) between the IMN and non-IMN groups (p < 0.05), but there was no significant difference in serum creatinine and urea concentrations (Table 1).

**Table 1:** Clinical characteristics of patients.

	Normal range	All patients (n = 135)	IMN (n = 68)	Non-IMN (n = 67)	P-value
Age (years)		48 (36–57)	49.5 (41.5–56)	47 (32–58)	0.28
Sex (female/male)		62/73	27/41	35/32	0.144
Serum total protein (g/L)	58–80	54.62 (46.25–61.65)	50.21 (43.42–57.71)	58.63 (50.41–65.54)	< 0.001
Serum albumin (g/L)	38–60	29.91(22.20–34.93)	28 (21.31–32.46)	33.24 (25.30–36.38)	0.003
24-h proteinuria (g/24 h)	0–0.25	3.26 (1.89–7.68)	5.29 (2.64–9.41)	2.32 (0.88–4.08)	< 0.001
Serum creatinine (μmol/L)	44–88	72.82 (53.67–93.38)	69.83 (52.56–88.01)	75.58 (58.52–105.52)	0.073
eGFR (ml/min per 1.73 m^2^)	≥ 90	102.82 (69.55–129.04)	106.1 (85.89–136.34)	96.57 (55.83–125.42)	0.028
Urea (mmol/L)	2.3–7	5.26 (4.16–7.44)	4.93 (3.72–7.03)	5.41 (4.28–8.05)	0.159
Cystatin C (mg/L)	0.65–1.09	1.17 (0.91–1.51)	1.07 (0.85–1.33)	1.22 (0.97–1.7)	0.024

Continuous variables are presented as median (interquartile ranges).

*IMN* idiopathic membranous nephropathy, *eGFR* estimated glomerular filtration rate.

### Characterization of QD-mAb probes

We designed hydrophilic core–shell CdSe/ZnS QDs bound to mAb. Supplementary Fig. 1a shows the absorption (356 nm) and photoluminescence spectra (615 nm) of water-insoluble QDs. To change the hydrophobic properties of CdSe/ZnS QDs and enable their transfer to aqueous solution, QDs were encapsulated with amphiphilic polystyrene particles. TEM images of the QDs encapsulated with amphiphilic polystyrene particles are shown in Supplementary Fig. 1b. CdSe/ZnS QDs were evenly bound to the surface and inside of polystyrene particles. Prepared QDs exhibited a narrow particle-size distribution and highly homogeneous monodispersity, with a mean particle size of ~ 305 nm. We identified the effect of different coupling ratios between hydrophilic QDs and mAb on test performance. Specifically, a QDs:mAb coupling ratio of 1:0.2 had the strongest T/C fluorescence; therefore, we selected this ratio for coupling hydrophilic QDs to antibodies (Supplementary Fig. 1c).

After optimizing the coupling conditions, we determined the sizes and fluorescence spectra of the QD-antibody and hydrophilic CdSe/ZnS QDs (Supplementary Fig. 1d,e). The fluorescence peak of the QDs-mAb solution did not differ significantly from that of hydrophilic QDs with respect to shape and position, although the fluorescence intensity declined as uncoupled components remained in the supernatant during this process (Supplementary Fig. 1d). After antibody labeling, the hydrodynamic analysis revealed that QDs-mAb size increased from 305 to 345 nm (Supplementary Fig. 1e), indicating the successful formation of the QDs-mAb conjugate. The zeta potential of the QD-ICA was then detected to determine conjugate stability. Generally, a zeta potential >  + 30 mV or <  − 30 mV indicates stability in solution^22^. We determined a zeta potential of − 55.2 mV for the conjugate, suggesting that its carboxyl group can provide sufficient colloidal stability in an aqueous solution (Supplementary Fig. 1f).

### Optimization of the QDs-mAb probes

Next, we analyzed the stability and optical properties of the QDs-mAb probes under physiological conditions. We found that the QDs-mAb remained stable at a pH range of 6.0–9.0, with an optimal pH of 7.5 (Supplementary Fig. 2a). The analysis of PL intensity showed that the fluorescence intensity of the QDs-mAb probe was the strongest in the MES buffer (Supplementary Fig. 2b). Therefore, we evaluated the effect of PL intensity in the presence of MES buffer (pH 7.5) under conditions of six different ionic strengths and found that the QDs-mAb in MES buffer (0.005 M, pH 7.5) exhibited the highest fluorescence intensity; thus, this was selected as the diluent for the subsequent analyses (Supplementary Fig. 2c).

### Optimal proportions of coated PLA2R and QDs-mAb

We performed a checkerboard titration test to determine the optimal dilution ratios of the QDs-mAb probe and coated-antigen concentrations. The measured fluorescence intensity of varying concentrations of PLA2R and QDs-mAb demonstrated that the conditions were optimum at a QDs-mAb dilution of 1:100 and a coated antigen concentration of 0.5 mg/mL (Table 2).

**Table 2:** Optimized conditions for the coated PLA2R and the QD-labeled antibody.

Coating PLA2R	0.5 mg/mL				1 mg/mL				2 mg/mL			
Standard serum (RU/mL)	2	20	100	500	2	20	100	500	2	20	100	500
**QDs-mAb dilution rate**												
1:200	247	1322	4376	10,175	306	1017	2539	6878	295	1185	2869	6769
1:100	249	2463	5668	10,477	292	2453	4400	10,533	258	2061	4489	8470
1:50	261	2342	5612	10,522	295	1222	2637	5553	337	1283	2721	5667

Values are fluorescence at 615 nm, determined in the checkerboard titration test.

### Determination of incubation time

**Figure 2:**
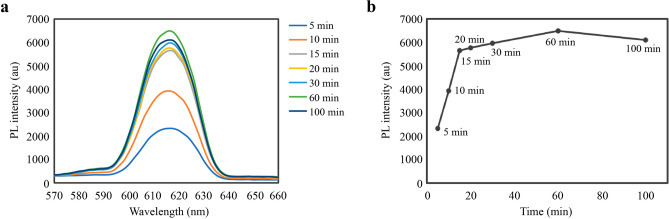
Optimization of the assay time. (**a**) Detection of fluorescence intensity under different fluorescence-developing times. (**b**) Identification of the optimal fluorescence-developing time. *PL* photoluminescence.

To determine the optimal time required for fluorescence development, we measured fluorescence intensities at different times. The results showed that the optimal fluorescence-development time to establish dynamic equilibrium between the coated antigen and the QDs-mAb was 15 min (Fig. 2). As the QDs-mAb tends to be released from the immune complex, the maximum fluorescence intensity at 60 min began to decrease.

### The performance of QD-ICA

Limit of blank (LoB) was used to determine the highest concentration of anti-PLA2R antibodies detected in the blank samples, and limit of detection (LoD) was used to determine the lowest concentration that the method could reliably detect to determine the presence or absence of anti-PLA2R antibodies. The LoB (the 95th percentile of the detected data) was 1.94 RU/mL, and the LoD value (LoD = LoB + 1.645 SD) was 2.86 RU/mL, with a broader linear range of the sample between 3 and 1500 RU/mL. The precision and matrix effect of QD-ICA were investigated by recovery experiment. The inter-batch recovery rate was 93.3–105.4%, and the intra-batch recovery rate was 96.2–102.7% (Table 3). The evaluation of serum matrix effects showed that the recovery rate of three samples was 96.30–104.63%, indicating that no significant matrix effect was observed with this method (Table 4).

**Table 3:** The precision of QD-ICA at three spiked levels.

Spiked concentration (RU/mL)	Inter‐assay			Intra‐assay		
	Mean	Recovery rate (%)	CV (%)	Mean	Recovery rate (%)	CV (%)
20	21.08	105.4	3.2	20.54	102.7	4.6
40	38.6	96.5	5.3	39.37	98.4	2.2
80	74.65	93.3	2.9	76.96	96.2	3.5

Spiked concentration is defined as the final concentration of the samples added to calibration serum.

*CV* coefficient of variation.

Recovery rate (%) = (recovery concentration/spiked concentration) × 100%.

CV (%) = (standard deviation/mean) × 100%.

**Table 4:** Evaluation of the serum matrix effects.

	Sample A	Sample B	Sample C
Positive sample concentration (RU/mL)	102.5	495.8	1011.2
Added positive sample concentration (RU/mL)	20.5	99.16	202.24
Detection concentration (RU/mL)	24.85	101.17	198.16
Recovery concentration (RU/mL)	21.45	97.77	194.76
Recovery rate (%)	104.63	98.60	96.30

Positive sample concentration is defined as the concentration of samples that were positive for PLA2R antibodies, as detected by ELISA (EUROIMMUN).

The detection concentration of negative serum was 3.4 RU/mL.

Recovery concentration (RU/mL) = detection concentration of samples—detection concentration of negative serum.

Added positive sample concentration (RU/mL) = positive samples concentration × added sample amount.

Recovery rate (%) = recovery concentration / addition concentration × 100%.

### QD-ICA immunoassays targeting the anti-PLA2R antibody

**Figure 3:**
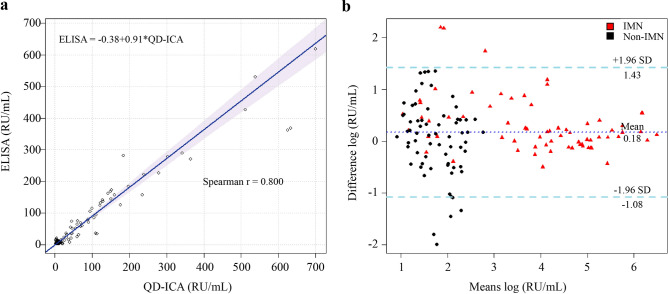
Consistency analysis for QD-ICA and ELISA. (**a**) Comparison of Passing and Bablok regression analyses for the two assays (regression equation was Y = 0.91x − 0.38, the Spearman correlation coefficient r was 0.8). (**b**) Bland–Altman plot of log-transformed difference against the mean of QD-ICA and ELISA. Dashed lines represent 1.96 ± standard deviation (the confidence interval of repeated measures is 95%).

**Figure 4:**
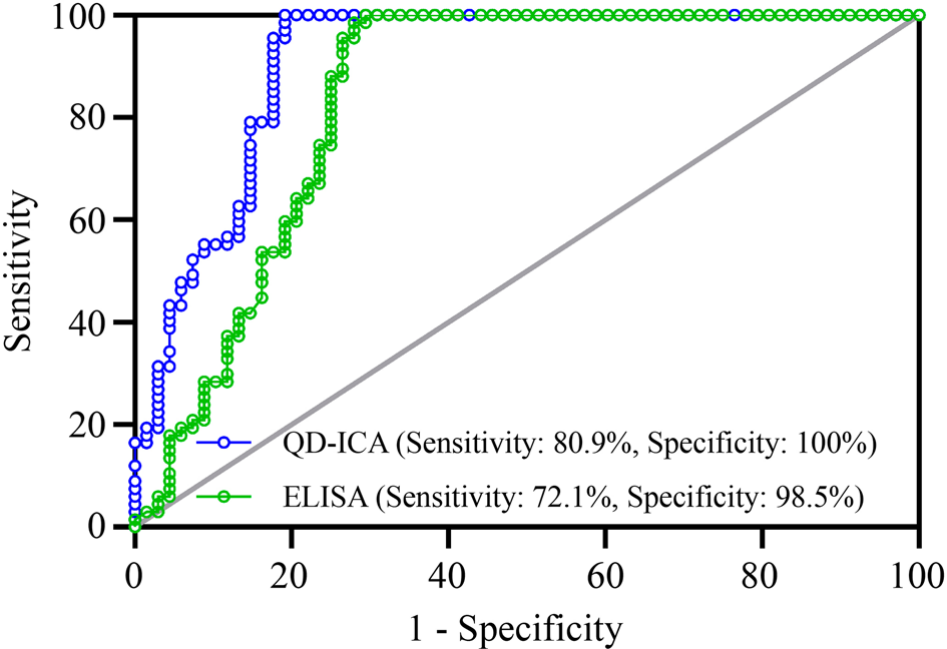
Receiver operating characteristic curve analysis of the two assays. *PL* photoluminescence.

ELISA (EUROIMMUN) was used to compare the QD-ICA results, generate scatter plot, and establish statistical regression equations. Human serum was diluted (1:100) with sample buffer according to Dähnrich et al.^16^. All serum samples were tested in triplicates, and the average values were used for further analysis. The Passing-Bablok regression equation was Y = 0.91x − 0.38, and Spearman's correlation coefficient r is 0.8 (Fig. 3a). The Bland–Altman plot (Fig. 3b) showed that the two assays are in good agreement with no obvious bias. The anti-PLA2R antibody sensitivity and specificity detected using QD-ICA (80.9% [95% confidence interval (CI) 69.2–89.0%] and 100% [95% CI 93.2–100.0%], respectively) exceeded those of ELISA (72.1% [95% CI 59.7–81.9%] and 98.5% [95% CI 90.9–100.0%], respectively). Samples that were positive for anti-PLA2R antibodies confirmed using ELISA were also positive in the QD-ICA test. However, anti-PLA2R antibodies were not detected in six patients with IMN via ELISA. The ROC analysis showed the AUC of 91.1% [95% CI 85.9–96.3%] for QD-ICA and 83.9% [95% CI 76.5–91.2%] for ELISA (p < 0.01) (Fig. 4). ROC analysis indicated that the optimal cut-off point for the QD-ICA was 18.18 RU/mL.

A comparison of the analytical performance of different methods for detecting the anti-PLA2R antibody is shown in Table 5. Conventional western blotting, IIFA assays, and commercial CBA-IFA assay are all semiquantitative analyses of anti-PLA2R antibodies. Although the sensitivities of these methods were comparable to those of quantitative detection methods, their complicated and time-consuming nature would hinder their application in clinical laboratories. Moreover, these methods were less reliable as the results depended on the observer's interpretation. ELISA, ALBIA, TRFIA, and LIPS quantitative detection methods improved detection speed, but the detection times remained longer than 2 h, making them unsuitable for use as POCT tests. Additionally, ALBIA and LIPS assays were less sensitive than the other methods listed in Table 5. The ChLIA method had similar sensitivity and specificity as the developed method here, but the detection system relied on expensive large-scale equipment, which was not applicable in community service points or areas with insufficient medical resources. These results showed that compared to other methods used to detect the anti-PLA2R antibody, QD-ICA was both rapid and highly sensitive.

**Table 5:** Comparison of the analytical performance of different methods for detecting the anti-PLA2R antibody.

Method	Origin cohort	Test characteristics	Patients	Linearity range	LoD	Sensitivity (%)	Specificity (%)	Cut-off	Time	References
Western blot	China	Semiquantitative	IMN (n = 60), lupus-associated MN (n = 20), (HBV)-associated MN (n = 16), tumor-associated MN (n = 10)			81.7	89		> 10 h	^28^
IIFA	Germany	Semiquantitative	IMN (n = 100), SMN (n = 17), non-MN (n = 90), healthy controls (n = 153)			52	100	1:10	> 1 h	^11^
CBA-IFA	Germany/USA	Semiquantitative	IMN (n = 157), non-MN (n = 41), SLE (n = 26), GPA (n = 25), healthy controls (n = 50)			63.7	99.3	1:10	> 10 h	^18^
ALBIA	Germany/USA	Quantified	IMN (n = 157), non-MN (n = 41), SLE (n = 26), GPA (n = 25), healthy controls (n = 50)			66.9	97.9	592 MFI	> 3 h	^18^
ELISA	France/Sweden	Quantified	IMN (n = 155), glomerular diseases controls (n = 154)			73.5	100	20 RU/mL	> 2 h	^21^
TRFIA	China	Quantified	IMN (n = 69), SMN (n = 9), other glomerulonephritis (n = 94), healthy controls (n = 286)	0.02–340 mg/L (measurement range)	10^−18^ mol/L	71.01	100	0.91 mg/L	> 2 h	^24^
LIPS	Boston	Quantified	MN (n = 45), other nephropathy controls (n = 18), healthy controls (n = 8),			53.3	100	47,268 LU^a^ and 33,242 LU^b^	> 2 h	^20^
ChLIA	France/Sweden	Quantified	IMN (n = 155), glomerular diseases controls (n = 154)			83.9	99.4	10 CU/mL	> 20 min	^21^
QD-ICA	China	Quantified	IMN (n = 68), SMN (n = 9), other nephropathy controls (n = 58)	3–1500 RU/mL	2.86 RU/mL	80.9	100	18.18 RU/mL	15 min	This study

*IIFA* indirect immunofluorescence assay, *SLE* systemic lupus erythematosus, *GPA* granulomatosis with polyangiitis, *CU/mL* chemiluminescent units per milliliter.

^a^Gaussian luciferase reporters.

^b^Nano-luciferase reporters.

## Discussion

Detection of the anti-PLA2R antibody significantly improved early diagnosis and appropriate clinical treatment of IMN. Here, we developed a novel POCT test (QD-ICA) for the quantitative detection of PLA2R autoantibodies and compared its performance with commercially available ELISA. Compared to other assays targeting anti-PLA2R antibody detection, QD-ICA was highly sensitive and faster, with an observed clinical sensitivity and specificity of 80.9 and 100%, respectively (both exceeding ELISA results). Furthermore, QD-ICA LoD was as low as 2.86 RU/mL, further demonstrating the superiority of QD-ICA to ELISA.

The titers of anti-PLA2R antibodies change throughout the IMN-treatment period^14^. Increasing levels of PLA2R autoantibodies indicate the exacerbation of symptoms, whereas decreased levels correlate with disease remission^23^. Therefore, it is necessary to detect autoantibodies quantitatively. Previous studies used western blotting, which requires electrophoresis and immunoblotting, as well as the use of naturally purified glomerular glycoprotein or extracts from cells overexpressing PLA2R, which may not be available in conventional clinical laboratories. Similarly, immunofluorescence testing methods are semiquantitative and observer-dependent, making them unsuitable for clinical testing.

Several methods have been developed to quantify PLA2R autoantibodies based on recombinant human PLA2R, including CBA-IFA, ELISA, ALBIA, TRFIA, LIPS, and ChLIA^18,20,21,24^. Positivity rates for anti-PLA2R detection using QD-ICA (80.9%) were similar to those reported previously for the other assays [ELISA (50.0–71.8%)^25,26^, ALBIA (51.5–66.9%)^18,27^, TRFIA (71.0%)^24^, LIPS (53.3%)^20^, and ChLIA (83.9%)^21^]. Although the positivity rates with western blotting (53.0–81.7%)^28,29^ and CBA-IFA (48.0–82.3%)^30,31^ are also comparable to those of quantitative assays, the semiquantitative nature of these methods hinders their clinical application.

The EUROIMMUN ELISA is currently widely used in clinical laboratories. The cut-off value as per the manufacturer instructions is recommended at 20 RU/mL, and values exceeding this are considered positive and those < 14 RU/mL are considered negative. However, a value between 14 and 20 RU/mL represents an uncertain outcome. Moreover, 20 RU/mL results in specificity ranging from 89.7 to 100%^18,25,32–36^. Some studies used different customized thresholds (e.g., 2, 2.6, or 14 RU/mL) to increase sensitivity^18,33,34,36^, although these sometimes adversely affected specificity. In the present study, ROC analysis revealed an optimal cut-off value of 17.84 RU/mL with ELISA, which resulted in maximum sensitivity and specificity of 72.1 and 98.5%, respectively, which was still lower than the results obtained using the QC-ICA. Bobart et al. found that cases tested using ELISA with results ranging from 2 to 20 RU/mL and positive via IFA were confirmed as MN via biopsy. However, 57.5% of cases tested using ELISA with results ranging from 2 to 20 RU/mL and with negative values via IFA were confirmed as MN via biopsy^37^. Therefore, Bobart et al. suggested that samples with ELISA results ≥ 2 RU/mL and ≤ 20 RU/mL should be confirmed via IFA before performing MN-related biopsies to improve the diagnostic sensitivity. Among our 68 patients with IMN, confirmed using renal biopsy, samples that tested positive for anti-PLA2R antibodies using ELISA were also positive in the QD-ICA test. However, anti-PLA2R antibodies from six patients with IMN were not detected with ELISA, suggesting that QD-ICA was more sensitive than ELISA and did not require a confirmation test.

QD-ICA is a subset of lateral flow assay-based POCT devices category and is performed over a strip that consists of a sample pad, marker-binding pad, absorption pad, and NC membrane. Compared with ELISA, this method is easy to operate and has low operating costs^38^. The advantages offered by QD-ICA, including simplicity, user-friendliness, cost-effectiveness, the ability to quantify the results, and high reagent stability, would be a breakthrough for IMN diagnosis. QD-ICA exhibited improved sensitivity due to its reliance upon labeling using aqueous QDs rather than macromolecular enzymes, thereby allowing more efficient labeling. Additionally, the use of aqueous QDs increases the Stokes shift between the excitation and emission wavelengths, thus, effectively circumnavigating environmental interference arising from a relatively long fluorescence-emission period. There were some limitations to this study. All patients enrolled in the study were from a single center, and a limited number of cases were included in this study. In addition, only the performance of QD-ICA was compared with that of ELISA for the detection of PLA2R autoantibodies. To further determine the potential advantages and disadvantages of QD-ICA, additional detection studies are needed for comparative analysis.

In summary, this study constructed a rapid, quantitative, and highly sensitive anti-PLA2R antibody detection assay using a CdSe/ZnS-based immunosorbent assay. The novel QD-ICA has the advantages of faster detection time, fewer steps, wider testing scenarios, and greater overall feasibility for detecting PLA2R autoantibodies compared to ELISA. These findings suggest that QD-ICA can improve IMN diagnosis and monitoring of patient response to treatment. We will further optimize our assay and use a larger number of clinical samples to confirm the performance of the assay, hoping to achieve clinical implementation.

## Methods

### Materials and instruments

Cadmium oxide (CdO, 99.99%), selenium (Se, 99.99%), zinc oxide (ZnO, 99.99%), sulfur (S, 99.98%), oleic acid (OA, 90%), 2-(*N*-morpholino) ethanesulfonic acid (MES), 1-octadecene (ODE, 90%), and monoethanolamine were obtained from Sigma-Aldrich (St. Louis, MO, USA). Sodium hydroxide (NaOH, 96.0%), hydrochloric acid (HCl, 37%), Na_2_HPO_4_, boric acid (H_3_BO_3_, 99.8%), sodium borate (Na_2_B_4_O_7_·10H_2_O, 99.5%), NaCl, KCl, Tris, Hepes, Tween-20, Na_2_CO_3_, NaHCO_3_, KH_2_PO_4_, PEG6000, sucrose, TritonX-100, and polystyrene particles were purchased from Sangon Ltd (Shanghai, China). N-Hydroxysulfosuccinimide (sulfo-NHS), N-(3-dimethylaminopropyl)-N′-ethylcarbodiimide hydrochloride (EDC) and bovine serum albumin (BSA) were purchased from Sigma-Aldrich. Mouse anti-human IgG mAb, the PLA2R recombinant antigen, and N-2,4-dinitrophenylated BSA (DNP-BSA) were purchased from Vazyme (Nanjing, China). Fluorescence spectra were measured using a fluorescence spectrofluorometer (Thermo Fisher Scientific, Waltham, MA, USA). Real-time dynamic light scattering signals of the QDs and the QDs-antibody probe were detected using a Zetasizer Nano ZS system (Malvern Panalytical, Malvern, UK). Selected mAbs and QD probes were dispensed on the sample pad using a gold-dispensing system (Jinbiao, Shanghai, China). Photoluminescence (PL) spectra of the control (C) line and test (T) line on the QD-ICA were recorded using the automatic fluorescence immunoanalyzer QD-S600 (Vazyme). All other reagents were purchased from Vazyme.

### Study participants

A total of 135 biopsy-confirmed nephrotic syndrome patients [IMN (68), SMN (9), and non-MN (58) with nephrotic syndrome (IgA nephritis (21), diabetic nephropathy (8), lupus nephropathy (7), focal segmental glomerular sclerosis (9), and immunocomplex-associated glomerulonephritis (13)] were enrolled from Shanxi Provincial People’s Hospital. The patients were diagnosed based on immunohistochemistry, light microscopy, and electron microscopy findings. Serum and urine samples were collected the day before renal biopsy (before initiating therapy). Serum samples from all patients were centrifuged for subsequent use. Before the interview and blood collection, written informed consent from adults and the consent of the parents of minors was obtained. All methods were performed in accordance with relevant guidelines and regulations. The study was approved by the Ethics Committee of Shanxi Provincial People’s Hospital. Participants were informed of the purpose of the study and their right to keep their information confidential.

### Establishment of QD-antibody conjugates

Hydrophobic core–shell CdSe/ZnS QDs were designed as reported previously, with appropriate modifications^39^ (“Supplementary Information”). QDs-mAb conjugates were prepared via a conventional EDC/NHS coupling reaction between the amino groups of the antibodies and carboxyl groups on the hydrophilic surface of QDs (Fig. 1). First, 50 μL of QDs (10 mg/mL) was dispersed in 450 μL of MOPS (0.02 M, PH 6.5), followed by activation with 3 μL (50 mg/mL) EDC and 3 μL (75 mg/mL) NHS. The activated QDs on ice were subjected to ultrasound for 5 min and collected by centrifugation at 17,000 × *g* at 18 °C for 15 min. The QDs sediment was dispersed in 500 µL MES (0.02 M, pH 6.0) and incubated with different amounts of mouse anti-human IgG mAb for approximately 30 min at room temperature (20 ± 5 °C). Next, we added 5 µL (10 mg/mL) of the reference protein DNP-BSA for quality control and incubated for 45 min at room temperature. The complex was blocked using 0.5% casein and terminated using 10% ethanolamine for 30 min at the same temperature. The terminated sediment was obtained by centrifugation and stored in 50 µL Tris buffer (5 mM, pH 7.5).

### Preparation of recombinant human PLA2R in HEK293 T cells

The extracellular domain of human PLA2R (NP_031392.3) was expressed in HEK293 T cells as described previously^11^. After culturing for ~ 1 week, a stable strain was collected for Ni^2+^-affinity chromatography purification. Recombinant human PLA2R was identified by performing nonreducing western blotting using serum positive for antibodies against PLA2R, as identified using ELISA (EA 1254-9601 G, EUROIMMUN AG, Lübeck, Germany). SDS-PAGE and western blotting were performed to detect the purity and confirm the molecular weight of the protein.

### Establishment of optimal proportions of coated PLA2R and QD-labeled antibody

A checkerboard titration test was performed to determine the optimal dilution ratios for the QDs-mAb probe and coated antigen concentration. The coated PLA2R antigen was diluted to different concentrations (0.5, 1, and 2 mg/mL) and fixed on a microplate in a volume of 100 μL, after which different concentrations of standard serum (2, 20, 100, and 500 RU/mL) were added to the corresponding wells and incubated at 37 °C for 30 min. The QDs-mAb probes were then diluted at different ratios [1:50, 1:100, and 1:200 (v/v)], followed by incubation at 37 °C for 30 min. Optical density was measured for each concentration.

### Design of the QD-based immunochromatography assay

The QD-based immunochromatography strip comprised the sample pad, marker-binding pad, absorption pad, and nitrocellulose (NC) membrane. The test strip was prepared using the indirect method. The steps are as follows. Sample pads (200 × 100 mm) made of fiberglass were saturated by placing them in Tris buffer (1 M, pH 7.5) containing 0.5% casein and 0.25% EDTA·2Na, followed by drying for 2 h at 45 °C. The conjugation pad was sprayed with QDs-mAb probe at a concentration of 4 µL/cm in Tris buffer (1 M, pH 7.5) containing 6% sucrose solution, 0.1% TritonX-100, and 0.05% polyvinylpyrrolidone K30 (PVPK-30), followed by drying at 45 °C for 18 h. Recombinant PLA2R and the anti-DNA-BSA antibody were sprayed onto the NC membrane (1 µL/cm) to form the T and C lines, respectively, and incubated overnight (12 h) at 37 °C in vacuo. Finally, all the parts were assembled and cut into a lateral immunochromatography strip (width: 3.6 mm) for subsequent detection. After addition, the serum diffused forward via capillary action. When passing through the marker pad and the NC membrane, the PLA2R autoantibody in the serum bound to QDs-mAb and recombinant human PLA2R to form a complex. The fluorescence signal was generated by exciting the QDs and was recorded by the automatic QD fluorescence immunoanalyzer QD-S600 (Vazyme). The PLA2R autoantibody level in the sample was linearly related to the intensity of the fluorescence signal within an appropriate range. After assembling the test strips, the coupling ratios of the QDs-mAb conjugate, dilution buffer, and fluorescence-development time were optimized to improve QD-ICA detection conditions (“Supplementary Information”).

### Analytical performance of QD-ICA

Presently, there is no reference standard for detecting anti-PLA2R antibodies; therefore, we determined the quantitative levels of QD-ICA using the EUROIMMUN ELISA kit (this kit has been registered by the FDA and is currently used in clinical laboratory testing). This ELISA kit was used to determine the PLA2R autoantibody concentration in the serum samples and select a sample (1556.84 RU/mL) with concentration in the upper limit of the linear range of the kit (1500 RU/mL). Several serum samples with low anti-PLA2R levels were selected and mixed to prepare a “negative sample” (< 4 RU/mL). The samples were diluted to different concentrations with the negative serum sample (< 4 RU/mL) as a calibrator to develop a standard curve. The level of the PLA2R autoantibody in the serum was calculated using the established standard curve.

The accuracy and precision of QD-ICA were evaluated as follows. Serum was added at different concentrations to the negative serum samples (< 4 RU/mL) to prepare a mixture with final concentrations of 20, 40, and 80 RU/mL. The spiked concentration of each PLA2R antibody was tested six times on the same day, and the test was repeated every 3 days in three batches. The average spike recovery rate of each spike concentration and the intra-batch and inter-batch differences of test strips were calculated. The serum matrix effect was evaluated as follows. Three samples with high anti-PLA2R levels were selected and defined sample A (102.5 RU/mL), sample B (495.8 RU/mL), and sample C (1011.2 RU/mL). The positive serum and 60 µL of distilled water were added to 240 µL of the negative serum sample (< 4 RU/mL), and the recovery concentration and recovery rate of different samples were calculated. LoB, LoD^40^, and the linear range were determined as described in the “Supplementary Information”.

### Statistical analysis

Statistical analysis was performed using SPSS (v.25; IBM Corp., Armonk, NY, USA). Continuous variables were represented as means (standard deviation) or median (interquartile ranges), and categorical variables were described as frequency rates or percentages. Mann–Whitney test was used to analyze continuous variables, and the chi-square and Fisher's exact tests were used to analyze categorical variables. The Passing-Bablok regression equation and coefficient of determination (R^2^) were calculated. The Bland–Altman plot of log-transformed difference was also used to describe the agreement between the two methods. The receiver operating characteristic (ROC) curve, area under ROC curve (AUC), and cut-off values of the QD-ICA were determined using clinical samples. A two-sided α of less than 0.05 was considered statistically significant.

## Supplementary Information

Supplementary Figure Legends.

Supplementary Figure S1.

Supplementary Figure S2.

Supplementary Information.

## Data Availability

The datasets analyzed in this study are available from the corresponding author on reasonable request.
