# Supplementary material for: Rapid, quantitative, and high-sensitivity detection of anti-phospholipase A2 receptor antibodies using a novel CdSe/ZnS-based fluorescence immunosorbent assay
Source: Sci Rep. 2021 Apr 22;11:8778. doi: 10.1038/s41598-021-88343-z (PMC8062494; doi:10.1038/s41598-021-88343-z)
Supplement: Supplementary file 1 — Supplementary Figure Legends. [file 41598_2021_88343_MOESM1_ESM.docx]

**Supplementary Figure 1**. Characteristics of the quantum dot–antibody conjugates. (a) Absorption spectra and photoluminescence spectra of the hydrophilic CdSe/ZnS quantum dots. (b) Transmission electron microscopy images of quantum dots encapsulated with amphiphilic polystyrene particles. (c) Determination of the coupling ratios between quantum dots and the antibody. (d) Photoluminescence spectra of the hydrophilic quantum dots and quantum dots–antibody conjugates. (e) Dynamic light scattering of the quantum dots–antibody conjugates and hydrophilic quantum dots. (f) Zeta potential curves of the quantum dots–antibody conjugates.

Abbreviation: PL, photoluminescence

**Supplementary Figure 2**. Evaluation of the fluorescence stability of quantum dots–antibody conjugates. (a) Fluorescence spectra of the quantum dots–antibody conjugates under different pH conditions and (b) in various buffers. (c) The photoluminescence intensity of the quantum dots–antibody conjugates in the optimal buffer (MES) at six different ionic strengths.

Abbreviation: PL, photoluminescence
