# Supplementary figures and images for: Rapid, quantitative, and high-sensitivity detection of anti-phospholipase A2 receptor antibodies using a novel CdSe/ZnS-based fluorescence immunosorbent assay
Source: Sci Rep. 2021 Apr 22;11:8778. doi: 10.1038/s41598-021-88343-z (PMC8062494; doi:10.1038/s41598-021-88343-z)

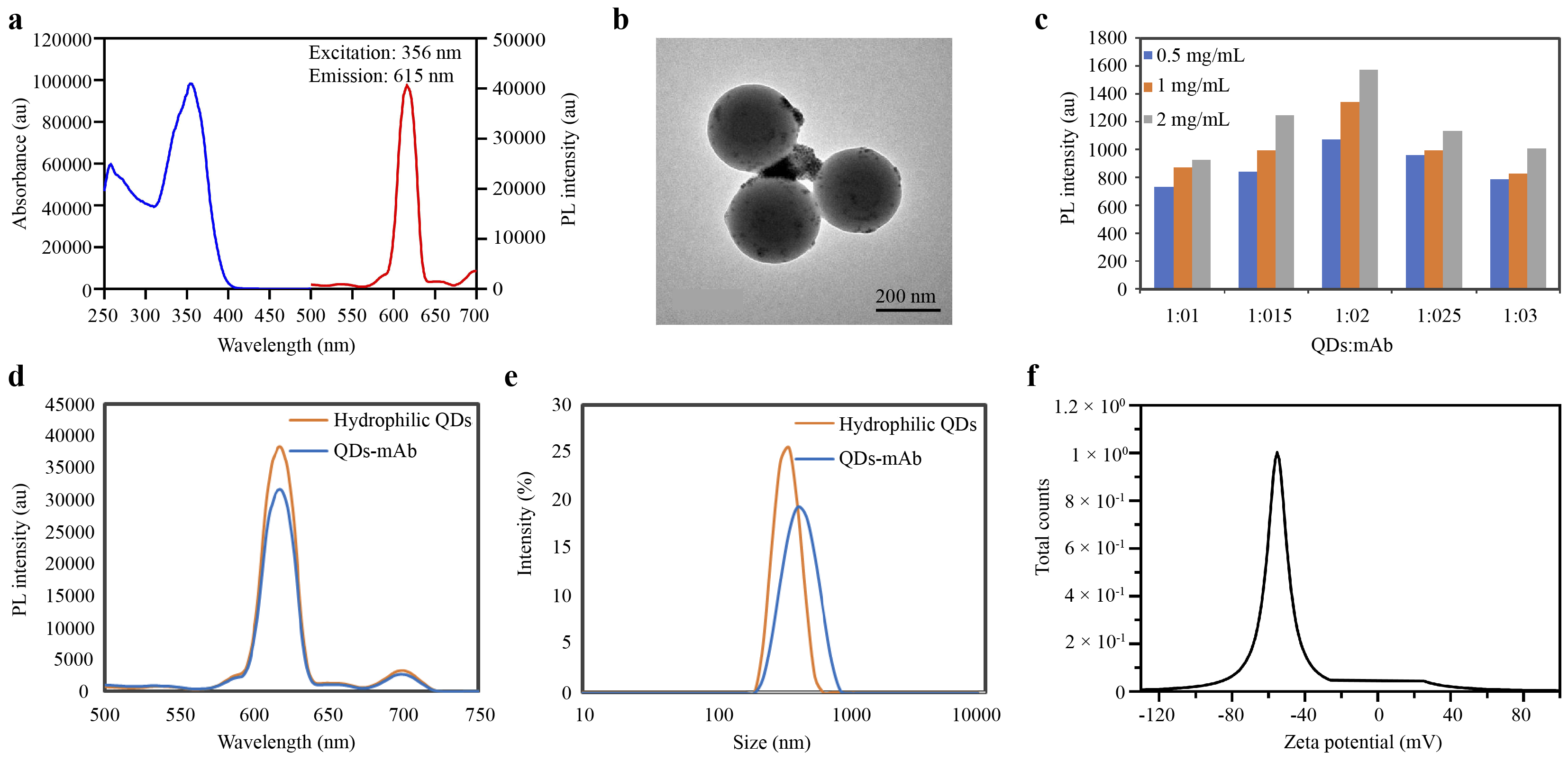

Supplement: Supplementary file 2 — Supplementary Figure S1. [file 41598_2021_88343_MOESM2_ESM.jpg]

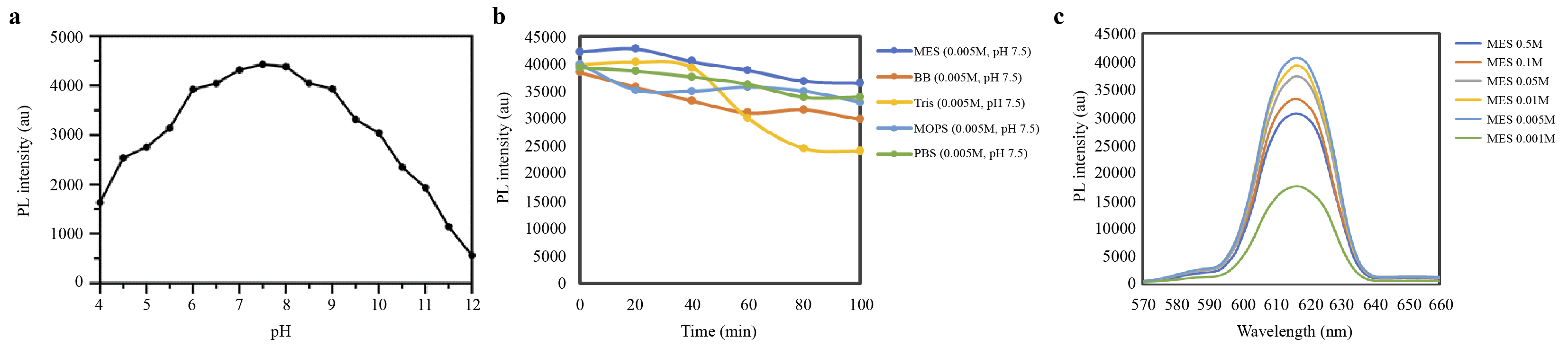

Supplement: Supplementary file 3 — Supplementary Figure S2. [file 41598_2021_88343_MOESM3_ESM.jpg]
