# Supplementary material for: Rapid, quantitative, and high-sensitivity detection of anti-phospholipase A2 receptor antibodies using a novel CdSe/ZnS-based fluorescence immunosorbent assay
Source: Sci Rep. 2021 Apr 22;11:8778. doi: 10.1038/s41598-021-88343-z (PMC8062494; doi:10.1038/s41598-021-88343-z)
Supplement: Supplementary file 4 — Supplementary Information. [file 41598_2021_88343_MOESM4_ESM.docx]

**Supplementary Information**

**Rapid, quantitative, and high-sensitivity detection of anti-phospholipase A2 receptor antibodies using a novel CdSe/ZnS-based fluorescence immunosorbent assay**

Chenxi Li^1^, Manyun Qian^2^, Qiaozhen Hong^3^, Xiaohong Xin^2^, Zichun Sun^4^, Yafeng Li^2*^, Bo Tang^1,4*^, Bing Gu^1,5*^

^1^Medical Technology School of Xuzhou Medical University, Xuzhou Key Laboratory of Laboratory Diagnostics, Xuzhou 221004, China

^2^Department of Nephrology, The Shanxi People’s Hospital, Shanxi Medical University, Taiyuan, Shanxi 030001, China

^3^Department of Laboratory Medicine, Quzhou Kecheng People's Hospital, Quzhou 324000, China

^4^Nanjing Vazyme Medical Technology Co. Ltd., Nanjing 210046, China

^5^Department of Laboratory Medicine, The Affiliated Hospital of Xuzhou Medical University, Xuzhou 221006, China

***Corresponding authors**

Bing Gu, Medical Technology School of Xuzhou Medical University, Xuzhou Key Laboratory of Laboratory Diagnostics, Xuzhou 221004, China; Department of Laboratory Medicine, Affiliated Hospital of Xuzhou Medical University, Xuzhou 221006, China; E-mail: binggu2015@xzhmu.edu.cn

Bo Tang, Medical Technology School of Xuzhou Medical University, Xuzhou Key Laboratory of Laboratory Diagnostics, Xuzhou 221004, China. Nanjing Vazyme Medical Technology Co. Ltd., Nanjing 210046, China; E-mail: tangbo@vazyme.com

Yafeng Li, Department of Nephrology, The Shanxi People’s Hospital, Shanxi Medical University, Taiyuan, Shanxi 030001, China; E-mail: Dr.yafengli@gmail.com

**Methods**

***Synthesis of aqueous CdSe/ZnS QD probes***

Hydrophobic core-shell CdSe/ZnS QDs were designed as reported previously, with appropriate modifications ^1^. Briefly, OA was used as a hydrophobic capping reagent for the QDs. The mixture (4 g) consisting of OA (1.5 mmol), CdO (0.08 g, 0.1 mmol), and ODE (10 mL) was loaded into a 50-milliliter round flask and heated to 280 ºC under an atmosphere of nitrogen. Next, 2 mL (0.5 mmol) of Se precursor stock solution was injected into the flask. Aliquots of CdSe QDs were collected at different time intervals and photoluminescence (PL) spectra were analyzed for each aliquot. Once target-sized CdSe QDs were obtained, the reaction mixture was cooled to room temperature (20 ± 5 °C). CdSe QDs (2.7 × 10^−7^ mol) in hexane were incubated at 100 °C for 30 min under nitrogen flow to remove hexane and other undesirable compounds. The solution was heated to 160 ºC under nitrogen flow to grow the ZnS shell. The calculated amounts of Zn and S precursors were added separately at 180, 200, 220, 240, and 250 ºC (the Zn precursor was obtained by dissolving ZnO in a mixture of ODE and OA at 310 ºC, whereas the S precursor was obtained by dissolving sulfur in ODE at 150 ºC). Once the reaction was completed, the reaction system was cooled to room temperature.

For transferring CdSe/ZnS QDs to aqueous solutions, QDs were encapsulated in polystyrene particles and used as probes for monoclonal antibody (mAb) labeling. Polystyrene particles and ZnSe/CdS QDs were dissolved in chloroform overnight at a molar ratio of 1:1.5 polystyrene particles/QDs. The mixture was rotary-evaporated at 45 °C to remove chloroform. The remaining QD sediments were dispersed in ammonia water (pH 8.5) by ultrasound. The ZnSe/CdS QDs were filtered using a 0.22-μm filter and centrifuged three times at 18,000 × *g* to remove excess oligomers. Transmission electron microscopy (TEM) was performed to capture the images of polystyrene particle-encapsulated ZnSe/CdS QDs.

***Optimization of the coupling ratio between QDs and mAb***

The QD-ICA test strip prepared previously (see “Design of the QD-based immunochromatography assay”) was used to identify the optimal coupling ratio between hydrophilic QDs and mAb. Different concentrations of a recombinant PLA2R antigen (0.5, 1, and 2 mg/mL) and QDs-mAb conjugates at different coupling ratios (1:0.1, 1:0.15, 1:0.2, 1:0.25, and 1:0.3) were coated on test strips. Calibrator serum (100 RU/mL) was added and incubated with QDs-mAb conjugates with different coupling ratios for the same duration. Under excitation and emission wavelengths of 356 and 615 nm, respectively, the T/C fluorescence of QDs-mAb conjugates with different coupling ratios was measured simultaneously (10–20 min).

***Optimization of QD-ICA***

Coupling ratios of the QDs-mAb conjugate, dilution buffer, and fluorescence-development time were analyzed to identify the optimal QD-ICA detection conditions. We tested different pH (4–12) conditions to determine the stability and optical properties of the QDs-mAb probes. At the optimal pH, we diluted QDs-mAb conjugates with MES, BB, Tris, MOPS, or PBS (each 0.005 M), and then measured PL intensity (at 5, 10, 15, 20, 30, 60 and 100 min) to determine the optimal dilution buffer and fluorescence development time for QDs-mAb probes. QDs-mAb conjugates diluted in buffer were incubated at 37 °C for 1 h before testing. After incubating the calibrator serum (500 RU/mL) with the recombinant PLA2R antigen for 30 min in 96-well plates, the QDs-mAb (secondary antibody) was added. The PL spectra were recorded for different incubation periods. Under the optimized detection conditions, the ideal regression coefficient of the standard curve was obtained.

***Analytical performance of*** ***QD-ICA***

The limit of blank (LoB) was used to determine the highest concentration of anti-PLA2R antibodies in blank samples, and the limit of detection (LoD) was used to determine the lowest concentration that could be reliably detected using this method to establish the presence or absence of anti-PLA2R antibodies ^2^. LoB and LoD were calculated in accordance with the methodology outlined in the CLSI EP17 document ^2^. Physiological saline (blank control) was tested 60 times, and the values for anti-PLA2R antibodies were recorded. If the detection values were normally distributed, the LoB threshold of the anti-PLA2R antibody corresponded to:

LoB = µB + 1.645 σB

where µB is the detection mean of blank control, σB is the standard deviation (SD) of blank control.

If the values exhibited asymmetric distributions, the LoB corresponded to the 95^th^ percentile of the detected data. Three samples containing low levels of anti-PLA2R antibodies were selected, and 12 replicates were performed to determine the SD for the LoD. The calculation formula was:

LoD = LoB + 1.645 σs

where σS is the SD of the low sample measurement population.

The linear range was determined by selecting a sample with anti-PLA2R antibody concentration (H) close to the upper limit of the linear range of the commercial kit (1,556.84 RU/mL according to ELISA; EUROIMMUN) and one with a low concentration (L) near the detection limit (3.12 RU/mL according to ELISA; EUROIMMUN). Calculations were made (L, 4L+1H, 3L+2H, 2L+3H, 1L+4H, and H) and the test was repeated for each dilution to calculate the mean value. Expected concentration levels were calculated to allow statistical analysis and acquisition of a regression equation and scatter plot.

**References**

1 Wu, F. *et al.* Multiplexed detection of influenza A virus subtype H5 and H9 via quantum dot-based immunoassay. *Biosens. Bioelectron.* **77**, 464-470; doi:10.1016/j.bios.2015.10.002 (2016).

2 Tholen, D. W. *et al*. Protocols for determination of limits of detection and limits of quantitation; approved guideline—second edition (CLSI EP17-A 24, 2004).
